# *In silico* modeling of spore inhalation reveals fungal persistence following low dose exposure

**DOI:** 10.1038/srep13958

**Published:** 2015-09-14

**Authors:** Reiko J. Tanaka, Neville J. Boon, Katarina Vrcelj, Anita Nguyen, Carmelina Vinci, Darius Armstrong-James, Elaine Bignell

**Affiliations:** 1 Department of Bioengineering, Imperial College London, London, UK, SW7 2AZ; 2 Department of Human Anatomy and Genetics, University of Oxford; 3 Barts and The London School of Medicine and Dentistry, Queen Mary University of London, London, E1 2AT, UK; 4 National Heart and Lung Institute, Faculty of Medicine, Imperial College London, London, UK, SW7 2AZ; 5 Institute of Inflammation and Repair, Core Technology Facility, University of Manchester, UK, M13 9NT

## Abstract

The human lung is constantly exposed to spores of the environmental mould *Aspergillus fumigatus*, a major opportunistic pathogen. The spectrum of resultant disease is the outcome of complex host-pathogen interactions, an integrated, quantitative understanding of which lies beyond the ethical and technical reach permitted by animal studies. Here we construct a mathematical model of spore inhalation and clearance by concerted actions of macrophages and neutrophils, and use it to derive a mechanistic understanding of pathogen clearance by the healthy, immunocompetent host. In particular, we investigated the impact of inoculum size upon outcomes of single-dose fungal exposure by simulated titrations of inoculation dose, from 10^6^ to 10^2^ spores. Simulated low-dose (10^2^) spore exposure, an everyday occurrence for humans, revealed a counter-intuitive prediction of fungal persistence (>3 days). The model predictions were reflected in the short-term dynamics of experimental murine exposure to fungal spores, thereby highlighting the potential of mathematical modelling for studying relevant behaviours in experimental models of fungal disease. Our model suggests that infectious outcomes can be highly dependent upon short-term dynamics of fungal exposure, which may govern occurrence of cyclic or persistent subclinical fungal colonisation of the lung following low dose spore inhalation in non-neutropenic hosts.

Annually, more than 2,000,000 human lives are lost to invasive fungal infections with the vast majority of fatal disease caused by lung-dwelling pathogens^1^. Opportunistic fungal infections are the outcome of a complex series of pathogen- and host-mediated activities^2^,^3^, our understanding of which has been mainly derived from a reductionist approach, using both *in vivo* and *in vitro* models of infection. Although an integrated understanding of regulatory mechanisms impacting disease outcomes is an essential prerequisite for designing and implementing novel antifungal interventions, this cannot be achieved by resorting solely to conventional experimentation due to the complex interactions among many entities involved. Moreover, the design and implementation of immunotherapeutic interventions demands significant improvement of current experimental modelling if the genetic and clinical heterogeneity amongst human patients is to be overcome. Recently the benefits of combining iterative mathematical modelling and experimental approaches to understand defective regulatory mechanisms leading to disease have been appreciated^4^,^5^,^6^. Mathematical modelling allows us to simulate many different conditions that may be difficult to test experimentally. Mathematically explicit and quantitative descriptions of the dynamics of the system, coupled with experiments, help us to produce and test new hypotheses^7^.

The widespread use of immunosuppressive conditioning regimens during cancer treatments and organ transplantation has heightened the threat of fatal fungal infections^1^. Among them, infections caused by the mould *Aspergillus fumigatus* are extremely problematic since they often evade detection in a timeframe conducive to effective management, and result in diseases having unacceptably high mortality, such as invasive pulmonary aspergillosis (IPA). *A. fumigatus* is a spore-forming mould whose spores (of 2–4 μM diameter) are so abundant in the air that inhalation of them is frequent in humans^8^. Innate immune mechanisms must therefore implement, in the human respiratory tract, constant surveillance and clearance of this pathogenic threat. *In vitro* studies reveal rapid (≤8 hours) macrophage-, and neutrophil-mediated, neutralisation of fungal spores by primary human phagocytes^9^,^10^. *A. fumigatus* is therefore an opportunistic pathogen, in the sense that immunocompetent hosts efficiently prevent invasive growth of inhaled spores and hyphae. However, altered host immune status, for example in hypersensitive or immuno-compromised individuals, promotes the development of various life-threatening diseases. The most serious of these, IPA, occurs in 30% of patients undergoing intensive chemotherapy for acute myelogenous leukemia–myelodysplastic syndrome and accounts for 43% and 19% of all invasive fungal infections in stem cell- and solid organ-transplant patients, respectively^11^,^12^. The annual European burden of chronic aspergilloses, occurring in the settings of chronic pulmonary disease, cystic fibrosis, and asthma, is predicted to exceed 2 million cases^13^.

Clearance of inhaled *A. fumigatus* spores requires the concerted activities of alveolar macrophages and neutrophils^14^,^15^. However, the relative potency of these cell subsets in effecting fungal clearance, and the mechanistic basis of their co-operative activity, remains unknown. Such information cannot be derived from *in vitro* experimentation, and cannot be readily quantified in whole animal models of disease. A major drawback of murine aspergillosis models is the requirement for large inocula (exceeding 10^5^ spores) to reproducibly initiate disease in immunocompromised hosts^16^. Compared with human infections, which are likely initiated from several tens (or fewer) of spores^8^, mice challenged with high numbers of spores have a much shorter duration of disease and a heightened severity of infection. Moreover, such excessive fungal challenge might simply overwhelm residual host defences, prompting physiologically irrelevant host responses. A further major problem is the lack of chronic aspergillosis models, for which sufficient understanding of relevant risk factors is lacking and whose lengthy timescales (months to years) prevent the ethical maintenance of murine cohorts large enough to reproducibly measure disease pathology.

In this study we use a mathematical modelling approach to address these challenges and to make quantitative predictions on the efficacy of neutrophils and macrophages in mediating fungal clearance at low dose fungal exposure. Significantly disparate short-term dynamics, in response to low- and high dose fungal exposure, emerged as a critical, counter-intuitive finding of the study which was experimentally validated thereby prompting new theories on the mechanistic basis of subclinical *A. fumigatus* persistence in the human lung. This work highlights the potential of mathematical modeling approaches for studying clinically relevant behaviors in experimental models of fungal disease.

## Results

### Model overview

**Figure 1:**
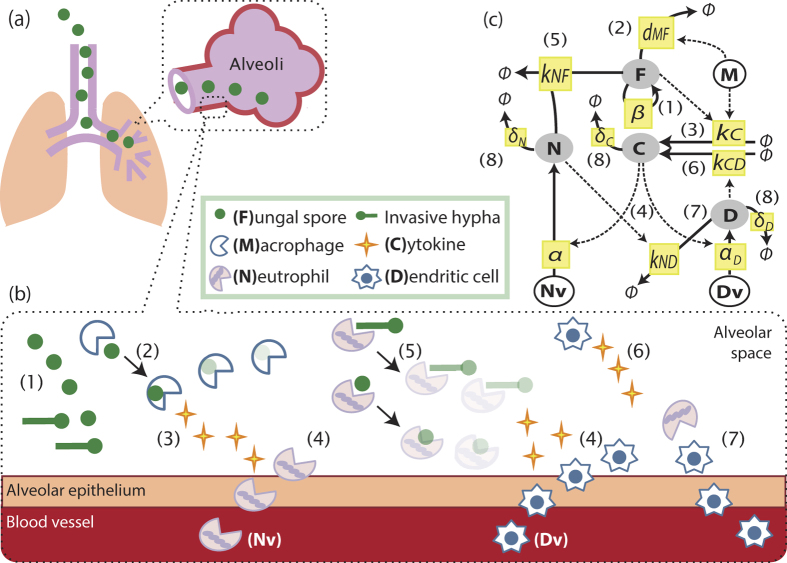
Host-pathogen interaction model. (**a,b**): Cartoon model of fungal exposure and killing for *A. fumigatus* and (**c**) a diagram for cellular interactions. Processes included in the model are: (1) Proliferation of fungal nuclei, leading to increased fungal burden (F), (2) Macrophage (M)-mediated fungal killing, (3) Production of cytokines (C) by macrophages, (4) Cytokine-induced recruitment of lung dwelling neutrophils (N) and inflammatory dendritic cells (D), from the neutrophil pool (Nv) or dendritic cell source (Dv), (5) Fungal killing by neutrophils, (6) Production of cytokines by inflammatory dendritic cells, (7) Neutrophil-mediated efflux of inflammatory dendritic cells, and (8) Degradation of cytokines, inflammatory dendritic cells and neutrophils (only shown in the diagram in (**c**)). (**c**) Variables in our model (F, C, N, D) are shown in shaded circles, whereas fixed parameters (M, Nv, Dv) are in black circles. Each process is shown by a yellow box with its parameter notation for the corresponding equation. Solid and dotted lines indicate the physical involvement of the process and the modulation of the rate of the process, respectively.

Inhalation of *A. fumigatus* spores leads to varying pathologic outcomes depending on the host immune status^17^. In healthy individuals fungal elements are thought to be cleared via appropriate collaborative activities of innate immune mechanisms, primarily by macrophages and/or neutrophils. We developed an ordinary differential equation model, comprising of eight biological processes (Fig. 1), which predicts and quantifies the short-term concerted actions of macrophages and neutrophils in response to fungal challenge and which ultimately determines the outcomes of fungal spore inhalation. A complete model description can be found in the Methods section. The model utilises parameter values derived from the extant literature on murine models of invasive aspergillosis and *in vitro* experiments (Table 1). Derivation of the parameters is detailed in Supplementary Information.

Inhaled *A. fumigatus* spores reach the alveoli of the human lung after physical (such as ciliary-mediated clearance) and mucosal (such as epithelial) activities have already taken effect (Fig. 1(a)), where they may persist and germinate to initiate pulmonary infection via invasive hyphae (elongated fungal cells). Our model (Fig. 1(b,c)), which addresses immediate responses to fungal challenge considers fungal proliferation (process (1) in Fig. 1(b,c)) in terms of conidial equivalents (CEs, are single haploid nuclei which proliferate in fungal spores and hyphae at a rate directly proportionate to that of fungal growth), the replication of which results in heightened fungal burden (F). Spores are phagocytosed and killed (2) by macrophages (M)^9^,^18^, with the concomitant release of cytokines (3)^19^ such as TNF-α. Cytokines (C) initiate recruitment (4) of available neutrophils (Nv) and recruitable inflammatory dendritic cells (Dv) from the blood vessels to the lung^20^. Recruited neutrophils (N) have potent anti-Aspergillus microbicidal activity and kill fungal elements via formation of a neutrophil-fungus complex (5)^21^. Recruited inflammatory dendritic cells (D) produce cytokines, such as TNF-α^20^, and are postulated as proponents of a positive feedback loop (6) for cytokine production. Neutrophils promote the maturation and outward trafficking (7) of inflammatory dendritic cells (iDCs), from the lung to draining lymph nodes. The absence of this activity in neutropenic individuals, due to fewer recruited neutrophils, promotes a marked accumulation of iDCs in the lungs of mice challenged with Aspergillus^22^. Degradation of cytokines, iDCs and neutrophils (8) are also included.

Our model assumes a constant number of available neutrophils (Nv), recruitable iDCs (Dv) and alveolar macrophages (M) in the immunocompetent mammalian host, while neutropenia is mathematically implemented as a 90% decrease in available neutrophils. Profound neutropenia (<100 neutrophils/μL, lasting more than 10 to 15 days) is a major risk factor for invasive mould infections in humans^23^. The model regards the number of resident alveolar macrophages as remaining constant, such that macrophages which phagocytose fungal spores retain viability. In contrast, and consistent with the known role of neutrophils in generating extracellular neutrophil traps (NETs), recruited neutrophils which interact with fungal elements are considered to no longer contribute to host activities. Nominal fungal inoculum (F_0_) is constrained to 10^6^ CEs, being that commonly used for murine challenge^16^.

Fungal exposure can be defined as an everyday occurrence which results from inhalation of fungal spores, where F_0_ is the number of spores which are deposited into the lung, and which are competent (after physical and mucosal activities have occurred) for proliferation. In the absence of published data on the relevance and/or magnitude of physical and mucosal activities, and assuming that such effects will be small compared to the magnitude of neutralising activities elicited by macrophages and neutrophils, we consider it reasonable to assume that F_0_ is equivalent to the experimentally administered fungal inoculum. Time zero (t = 0) is the time at which inhaled spores reach the lung, persistence is defined as fungal occupation of the lung with F > 1 CEs, following inhalation. Infection differs from persistence in that the pathogen burden is actively increasing (dF/dt > 0). The term ‘colonisation’ is used to describe situations where the lung is occupied by live fungus, which might include persistence, or infection, or both. Fungal containment is achieved when fungal increase is halted (dF/dt < 0). Fungal clearance is achieved when F < 1.

### Simulated outcomes of spore inhalation

**Figure 2:**
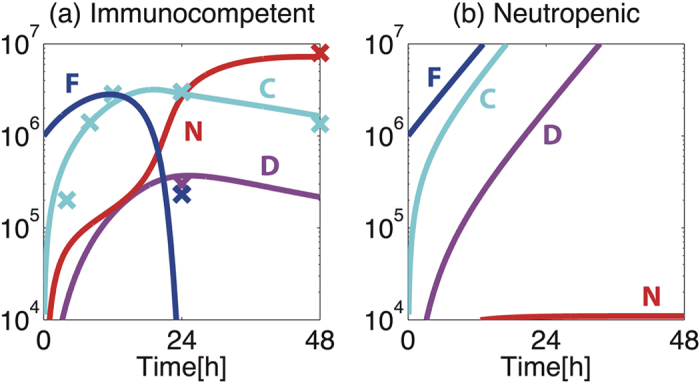
Simulated short-term dynamics of response to *A. fumigatus* challenge in (a) immunocompetent and (b) neutropenic hosts. Blue, cyan, red and pink depict fungal burden (F, [10^6^ CEs]), cytokines (C, [ng/ml]), recruited neutrophils (N, [10^6^ cells]) and recruited iDCs (D [10^6^ cells]), respectively. Solid lines represent model time-course and crosses represent the experimental data points used for parameter estimation^20^,^24^.

For initial simulations, we considered the impact of neutropenia upon the outcome of infection following fungal challenge with 10^6^ CEs. In immunocompetent hosts (Fig. 2(a)), a rapid increase in the level of cytokines (C), peaking at approximately 19 hours post-inoculation, precedes an increase in the levels of lung neutrophils (N) and iDCs (D). The fungal burden (F) is reduced to ≤5% of the initial value (F_0_) within 23 hours. Satisfied that these outcomes broadly concur with those commonly reported during murine challenge^24^,^25^,^26^, we simulated fungal exposure in a neutropenic host by *in silico* depletion of available neutrophils (Nv), a mathematical perturbation which resulted in considerable alteration of model behaviours (Fig. 2(b)). In the neutropenic host, recruited neutrophils remain close to basal levels, thereby permitting a rapid increase in fungal burden despite heightened iDC recruitment and cytokine production. Fungal burden increases rapidly within 24 hours of inoculation and fungal containment cannot be implemented.

This simple mathematical model of disease outcome in mammalian hosts bears a significant resemblance to the immunopathology of murine and human aspergilloses, since (i) simulated neutropenia is a risk factor for fungal proliferation, (ii) the *in silico* increase in cytokine production observed in the neutropenic host far exceeds that observed in immunocompetent subjects^20^ and (iii) iDCs increase in number in *in silico* neutropenic subjects. The latter two observations are consistent with a proposed positive feedback mechanism acting, in neutropenic hosts, to potentiate cytokine production via reduced efflux of TNFα-producing iDCs^22^.

### Relative contributions of macrophages and neutrophils to fungal killing

**Figure 3:**
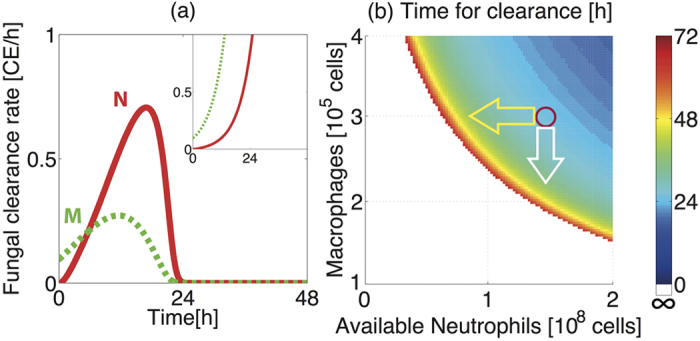
Impact of macrophage and available neutrophil titre upon clearance of *A. fumigatus* spores. (**a**) Rate of directly implemented fungal killing by neutrophils (*k*_NF_[N][F], red) and macrophages (*d*_MF_[M][F], green dashed) for immunocompetent hosts and neutropenic hosts (inset). (**b**) Time for clearance of fungal burden (10^6^ CE) for varying values of macrophages (M) and available neutrophils (Nv). Red circle indicates nominal values (with M = 0.3 × 10^6^ and Nv = 1.5 × 10^8^). Yellow and white arrows respectively depict the symmetric impacts of a decrease in Nv and a decrease in M. For small M and Nv (white area) fungal clearance is not achieved within 72 hours, leading to fungal persistence.

Our model assumes that reductions in fungal burden can be directly implemented by two mechanisms, namely through the microbicidal activity of macrophages, or via formation of neutrophil-fungus (NF) complexes which results in fungal killing. In order to derive new insight on the mechanism of host-mediated fungal killing by macrophages and neutrophils, we extracted and plotted the respective contribution of each mechanism from the model as a function of time, to effect a directly quantitative comparison of the relative contributions of macrophage (described by *d*_MF_[M][F] in the model equation) and neutrophil (described by *k*_NF_[N][F]) activities to fungal killing (Fig. 3(a)). In accordance with murine studies of aspergillosis^18^,^25^, this revealed that neutrophils predominate as the major agents of fungal killing in immunocompetent hosts. After the initial 6 hours of fungal exposure, neutrophil-mediated killing exceeds the rate of macrophage-mediated killing, achieving a maximal rate of killing within 17 hours of inoculation. Although the number of recruited neutrophils peaks at around 40 hours post-inoculation (Fig. 2(a)), F already begins to decrease considerably earlier (11.5 hours in Fig. 2(a)).

Macrophage- and neutrophil–mediated challenges to fungal survival are similarly observed in neutropenic hosts (Fig. 3(a), inset), however the contribution of neutrophils is much smaller than that observed in immunocompetent hosts. Thus, in the neutropenic case host defences are insufficient to contain rapid and continuous fungal proliferation, which necessitates monotonically increasing fungal killing rates for both macrophages and neutrophils (Fig. 3(a), inset).

Whilst the significance of macrophage activities rapidly becomes dwarfed by neutrophil-mediated killing, there is a notable period of time (first 6 hours post-inoculation) where macrophage activities dominate. This prediction is consistent with the hypothesis that macrophages may act as frontline defences against fungal challenge^8^. Whilst eliciting a lower magnitude than neutrophils of directly-implemented fungal killing in immunocompetent hosts (Fig. 3(a)), macrophage titre can nonetheless impact the efficiency of fungal clearance in our *in silico* simulation. This is evidenced by symmetric impacts of altered macrophage (M) and available neutrophil (Nv) titre on the time required for clearance of fungal burden (Fig. 3(b)), suggesting critical roles for macrophages, as well as for neutrophils, in fungal clearance. For example, a 40% decrease in Nv (yellow arrow) and a 28% decrease in M (white arrow) both result in an increase in the clearance time from 26 hours to 42 hours. This observation can, in part, be explained by the fact that macrophages are the main source of cytokine production in our model and are therefore crucial for recruitment of neutrophils to the lung.

Our simulations suggest that M-depleted hosts can achieve the same fungal clearance time as non-M-depleted hosts if N is heightened (implemented via Nv increase) in our model (Fig. 3(b)). This observation might underlie the previously reported, apparent non-involvement of macrophages in defence against *A*. *fumigatus* challenge in mice^25^,^27^ where clodronate-mediated macrophage depletion during murine aspergilloses has been found not to alter susceptibility to *A. fumigatus* challenge in whole animals, but to evoke the concomitant increase of neutrophil recruitment.

### Critical timing for neutrophil depletion: Cytokine-mediated neutrophil recruitment and fungal clearance

**Figure 4:**
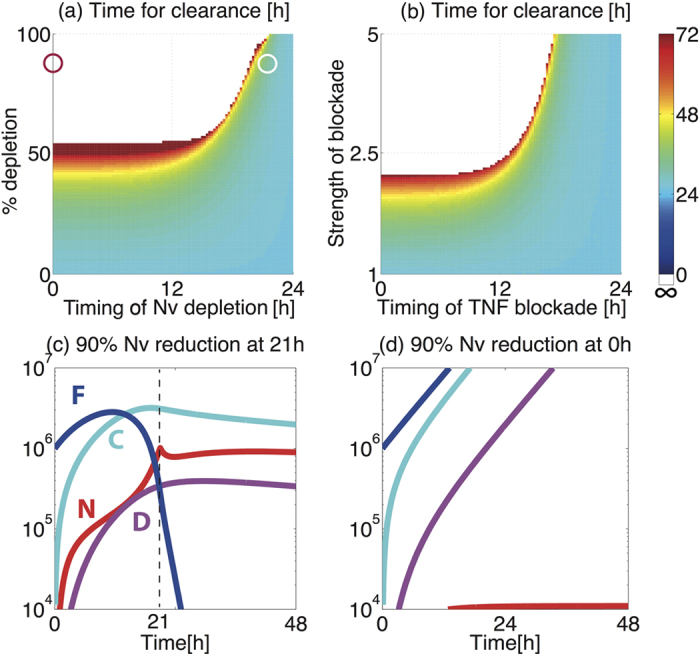
Timing-dependent effects of neutrophil depletion on fungal clearance time. (**a**) The time for fungal clearance (hours) with varied rate of Nv reduction introduced at different timings (hours post-inoculation). The red and white circles correspond to the conditions in (**c**) and (**d**). (**b**) The time for fungal clearance (hours) with varied rate of TNF-α blockade via decrease in cytokine levels introduced at different timings (hours post-inoculation). (c, d) 60% reduction of Nv (**c**) at the time of inoculation and (**d**) at 18 h post-inoculation.

Our *in silico* simulation further predicts a critical impact of the timing of neutrophil depletion upon time required for fungal clearance (Fig. 4(a)). For example, 90% reduction in Nv at later than 20 hours post-inoculation slows, but does not prevent, fungal clearance (Fig. 4(c)), while the same rate of Nv reduction at or before this critical time point leads to catastrophic impairment of fungal containment (Fig. 4(d)). Thus, there are a critical time-point, as well as a critical rate of reduction in Nv that determines whether fungal clearance is achieved. This observation suggests a critical early role for cytokine-mediated neutrophil recruitment in fungal containment, a theory supported by previously published murine experimentation^25^,^27^. Note that Nv appears in the first term *α*[*Nv*][C] of equation (3) which describes cytokine-mediated neutrophil recruitment.

Our model suggests quantitative mechanistic insight on this predicted timing-dependent effect of neutrophil depletion upon disease outcome. Nv depletion introduced at early phases, when the level of recruited neutrophils (N) is not yet high enough to contain F, causes F to increase and saturate. On the other hand, Nv depletion introduced at later phases, when N is already sufficiently high to contain F, does not prevent completion of fungal clearance, as F has already mostly been killed by N. Even if F was not cleared completely by the time of Nv depletion, previously recruited, but non-NF forming N will accumulate at the site, due to the low demand for NF formation. This result suggests that fungal clearance requires enough recruited neutrophils to combat fungal proliferation at early phases of fungal exposure (within 20 hours post-inoculation in the case of 90% Nv depletion), and further highlights the critical impact that short-term dynamics of antifungal immune responses can have upon outcomes of fungal exposure. Efficient fungal clearance requires a critical balance of the rate of fungal proliferation, which should be strong enough to produce enough cytokines to recruit a sufficient amount of N, but should be weak enough to avoid rapid saturation of F so that recruited neutrophils can achieve fungal clearance.

Similar timing-dependent effects on fungal clearance time are also observed during *in silico* M depletion (Supplementary Information, Fig S3), further supporting the crucial early role of macrophages for recruitment of neutrophils (Fig. 3), and during *in silico* TNF-α blockade implemented by reducing the cytokine levels (imposed in our model via fold-increase of the C degradation rate, δc) (Fig. 4(b)). These similarities suggest that less efficient neutrophil recruitment may be a potential mechanism for increases in fungal burden and mortality which occur during *in vivo* depletion of murine TNF-α^28^, and for the occurrence of invasive fungal infections in patients administered TNFα blockade therapy, which is used in several autoimmune diseases including inflammatory bowel disease and juvenile rheumatoid arthritis^29^.

### Simulated outcomes of low-dose fungal exposure

**Figure 5:**
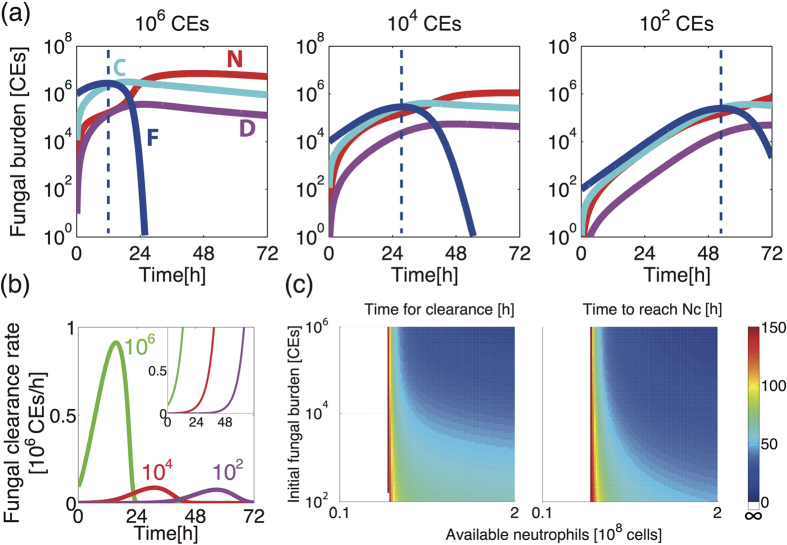
Simulation results for *A. fumigatus* inoculation with different initial (F_0_) dose (10^6^, 10^4^ and 10^2^ CEs) in immunocompetent and neutropenic hosts. (**a**) Fungal burden is contained at all inoculum sizes for immunocompetent hosts, with a noticeable delay in fungal containment for lower inoculum. Dotted lines indicate the start of the fungal containment (dF/dt < 0). (**b**) Rates of directly implemented fungal killing for different fungal inocula (10^6^, 10^4^ and 10^2^ CEs) for immunocompetent hosts and neutropenic hosts (inset). The area under the curve corresponds to the number of spores killed. (**c**) The time for fungal clearance and the time to reach Nc (and the F peak) demonstrate similar changes according to the changes in the inoculum size and the number of the available neutrophlis. The smaller the inoculum size or the number of the available neutrophils, the longer they take. For low Nv, fungal clearance is not achieved (white area). The colour bar indicates the time in hours.

Having established a model with a nominal parameter set capable of generating relevant behaviours, we next addressed the impact of a major disparity between murine models of fungal inoculation and human disease, namely the magnitude of fungal challenge. In immunocompromised murine hosts, inocula exceeding 10^5^ fungal spores are routinely used^16^ while IPA in humans is likely to be initiated by far fewer spores. Higher inocula may overwhelm residual immune defences and prompt physiologically irrelevant responses to fungal challenge. Additionally, the severity of fungal pneumonia arising from high dose exposure leads to rapid decline of the study cohort, with sacrifice of neutropenic hosts becoming necessary within 3 to 4 days. In order to interrogate model behaviours in response to lower fungal inocula, we mathematically titrated fungal burden from 10^6^ to 10^2^ spores and examined the short-term dynamics of response to fungal challenge in immunocompetent and neutropenic hosts (Fig. 5).

*In silico* titration of inoculation dose in immunocompetent hosts revealed a novel, and counterintutitve, prediction, whereby lower dose fungal challenges are contained less efficiently than high dose fungal challenges. In the case of an inoculum approximating 10^2^ spores, host-mediated fungal containment (defined as dF/dt < 0) starts at 53 hours post-inoculation, while it starts much earlier (11.5 hours) for challenges with 10^6^ spores (Fig. 5(a)). In the neutropenic case, regardless of inoculum size, fungal burden is never contained, although fungal burden increases more slowly for lower dose, than for high dose challenges. Moreover, our model simulation predicts that the higher dose inoculation results in not only earlier containment, but also in more spores being killed at earlier time points during the clearance process (Fig. 5(b)), suggesting stronger immune reactions triggered.

### A curative neutrophil threshold, Nc, must be met for effective implementation of fungal containment

The mechanistic basis for fungal persistence following low dose exposure can be explained by the requirement for a minimal number of recruited neutrophils to accumulate in order to achieve fungal containment. We define this as the ‘curative neutrophil threshold’ (Nc), which is 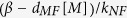 = 0.15 for the nominal case obtained by solving dF/dt = 0. When Nc is met or exceeded, neutrophil-mediated killing becomes more dominant than fungal proliferation, thereby resulting in fungal containment, i.e. a decrease in fungal burden (dF/dt < 0). Fungal containment starts and F reaches its peak value when N achieves Nc. If Nc is not met, as in the case of neutropenic hosts, fungal burden continues to increase (dF/dt > 0) and remains uncontained.

Our simulations demonstrate that the time required to achieve Nc (the time for F to reach its peak) and the time for fungal clearance both increase as inoculum size decreases and as the number of available neutrophils decreases (Fig. 5(c)). In the worst case scenario an inability to meet Nc leads to inability to achieve fungal clearance (white area). This observation suggests the importance of the initial proliferation of the fungal burden in prompting fungal clearance. Low dose fungal challenge evokes a milder immune response, resulting in slower acquisition of N, similar to the effect of reducing Nv, and thus extending the time required for fungal clearance.

From a biological viewpoint this prediction of slower containment for lower dose exposure can be rationalised if lower dose fungal challenge evokes a milder immune response, a hypothesis which garners favour if immunological tolerance of everyday spore exposure is accepted to occur. Indeed a plausible mechanistic basis for such tolerance (shedding of an immunoprotective hydrophobin–like protein RodA during spore germination) has previously been proposed^30^. From a clinical viewpoint such fungal persistence would provide a significant opportunity for inhaled spores to obtain a critical foothold in the airway, via latent occupation of the intracellular environment of epithelial or phagocytic cells to evade the host immune response, or by proliferating sufficiently enough to provoke an allergic response.

### Fungal persistence in immunocompetent murine hosts following low-dose fungal challenge

The mathematical model predicted, counter to expectations, that containment of fungal burden would be more slowly implemented in immunocompetent hosts challenged with lower dose fungal inocula (Fig. 5(a)) and that killing of higher dose inoculation proceeds more efficiently (Fig. 5(b)). In order to test the model predictions we performed two independent, and methodologically distinct, fungal challenge experiments.

**Figure 6:**
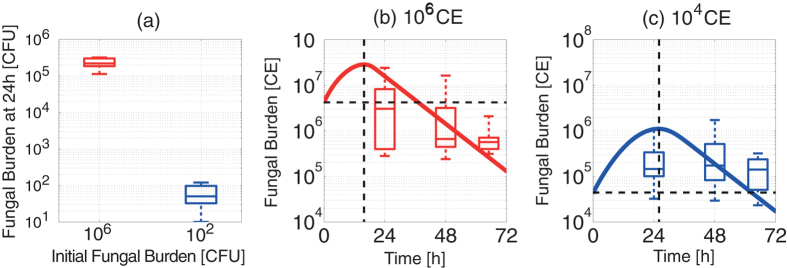
Experimental outcomes of spore inhalation in immunocompetent murine hosts with different *A. fumigatus* fungal inocula. (**a**) Viable colony forming units (CFUs) were enumerated from homogenised whole lungs (n = 5) at 24 hours after intranasal inoculation of 4 × 10^6^ and 400 spores. (**b–c**) Quantitation of conidial equivalents (CEs) by qPCR at 24 h, 48 h and 65 h after intranasal inoculation with (**b**) 4.25 × 10^6^ and (**c**) 4.25 × 10^4^ *A. fumigatus* spores (box plots) and the corresponding simulation results (lines). Dotted lines indicate the value of F_0_ (horizontal) and the start of fungal containment (dF/dt < 0) predicted by the corresponding model simulation (vertical).

To qualitatively test the model prediction prior to using increased numbers of infection cohorts and analysed time points, we challenged four groups of five immunocompetent animals with high (4 × 10^6^) or low (400) dose inocula of *A. fumigatus* spores and assessed fungal burden by enumeration of viable colony forming units (CFUs), per whole, homogenised, mouse lung, at 24 hours post-inoculation. The experimental results show that, for high dose inocula, fungal viable counts were reduced by 3.6–3.9 × 10^6 ^CFUs within the initial 24 hours of fungal challenge, compared to reductions of 280–390 spores in the case of low dose fungal challenge (Fig. 6(a) and Table S1). Thus, despite an inherent capacity to neutralise >10^6^ fungal spores during the 24 hours following initial fungal challenge, the host fails to eradicate a low dose fungal challenge in this time-frame. Although viable CFU counts provide an accurate read-out of persistence of viable fungus, a limitation of this approach is an inability to enumerate fungal proliferation in terms of nuclear doublings, since individual nuclei (or conidial equivalents) might number >100 in a single fungal hypha but still manifest as a single CFU when plated on solid culture media, thereby leading to underestimation of fungal burden.

To investigate the temporal dynamics of fungal burden at intermediate time points and inocula, we expanded the fungal challenge analysis, this time surveying fungal burden via PCR-mediated quantitation of conidial equivalents (CEs). Six groups of 5 immunocompetent animals were infected with high (4.25 × 10^6^) or low (4.25 × 10^4^) dose inocula of *A. fumigatus* spores, and fungal burden, per whole mouse lung, was quantified at 24, 48 and 65 hours post-inoculation. The experimental data (Fig. 6(b,c)) confirmed the mathematical prediction of fungal persistence, i.e. slower achievement of the peak F value, in low-dose challenge (Fig. 5(a)). For high dose fungal exposure the median net value of F becomes less than F_0_ from 24 hours onwards, suggesting that a peak value of F was achieved before 24 hours. On the other hand, the median fungal burden in lower dose infection does not decrease until at least 48 hours, suggesting that the peak of F was achieved after 24 hours. The experimental data validates general phenomenon that the mathematical model predicted, i.e. slower initial clearance of F following lower dose fungal exposure (Fig. 5(a)), and the experimental dynamics is similar to that of the model behaviour.

Note that our mathematical model assumes that all the cells and fungal spores are uniformly distributed in the lung. For lower dose exposure, the spatial sparcity of fungal elements might lead to a correspondingly lower probability of macrophage encounter. Precise quantitative comparison between the model and experimental results will require further experimental data and methodological innovation to overcome the sensitivity threshold of fungal enumerations of very low numbers of CEs. Future studies will address this possibility by implementation of flow cytometric assays for heightened accuracy of CE enumerations.

### Dichotomous outcomes of disease following low-dose *A. fumigatus* exposure: Cyclic or persistent fungal colonisation

**Figure 7:**
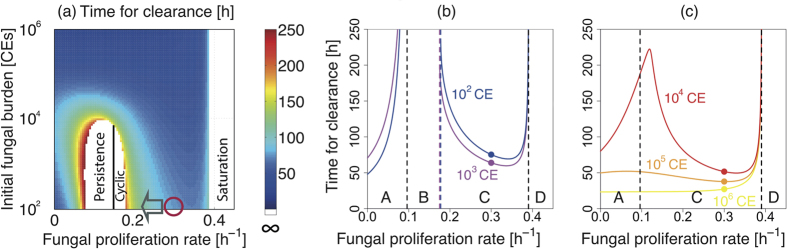
Potential impact of fungal proliferation rate (*β*) on the time required for fungal clearance. (**a**) The time required for fungal clearance (hours) with varied fungal proliferation rate (β) and initial fungal burden (F_0_). For some inoculum and proliferation rates, fungal clearance is not achieved within 250 hours (white area) and either cyclic or persistent colonisation is observed, depending on whether the value of the fungal proliferation rate is larger or smaller than the threshold value *β* = 0.15 (black vertical line). (**b,c**) The time required for fungal clearance (hours) for fixed initial fungal burdens. Low initial fungal burden case (10^2^ and 10^3^ CEs in (**b**)) has four distinct regions corresponding to different behaviors: (A) clearance mainly by macrophages, (B) oscillatory (persistent or cyclic) colonisation, (C) clearance mainly by neutrophils, and (D) saturation. High initial fungal burden case (10^4^, 10^5^ and 10^6^ CEs in (**c**)) has three regions (A, C and D), and does not exhibit oscillatory colonisation (B). The circles on the lines indicate the nominal value *β *= 0.28.

**Figure 8:**
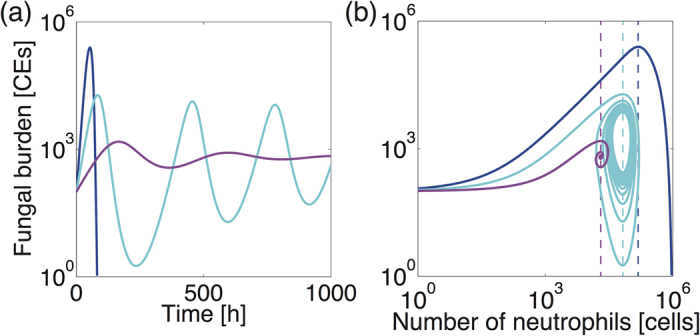
Potential for significant impact of short-term responses to fungal exposure upon infectious outcomes. Typical fungal burden dynamics (**a**) and the phase diagram for fungal burden against number of neutrophils (**b**) for fungal clearance (blue, *β = *0.28), cyclic colonisation (cyan, *β *= 0.175) and persistent colonisation (violet, *β *= 0.12), with F_0 _= 100. Fungal containment starts and F begins to decrease when the curative neutrophil threshold (Nc, dotted lines) is reached.

We next investigated the potential impacts of short-term fungal clearance dynamics on disease outcomes via mathematical simulations (Figs 7 and 8). This revealed a further counter-intuitive prediction for low dose fungal inoculation, whereby lowered rates of fungal proliferation (*β*) extend the time required for fungal clearance to be achieved. For example, in *in silico* hosts challenged with 10^2^ CEs, a decrease in the rate of fungal proliferation, from the nominal value of 0.28 h^−1^, to 0.22 h^−1^ (corresponding to an increase in fungal doubling time from 2.48 hours to 3.15 hours, respectively) results in an increase in the time required for clearance from 81 hours to 117 hours (grey arrow, Fig. 7(a)). Thus, in our mathematical model, a lower fungal proliferation rate evokes a milder immune response, resulting in slower acquisition of Nc, and extension of the time required for fungal containment.

Further *in silico* decrease of the fungal proliferation rate (*β*), for example to below 0.18 h^−1^ in the case of F_0 _= 10^2^, leads to incomplete curtailment of pathogen burden (white area, Fig. 7(a)), where subclinical chronic (either cyclic or persistent) colonisation is observed for low dose inocula (below 10^4^ fungal spores). In the case of cyclic colonisation (Fig. 8, cyan), an iterative cycle of incomplete fungal clearance, and proliferation of uncleared fungal elements occurs. In the case of persistent colonisation (Fig. 8, violet), a similar iterative cycle of fungal clearance and proliferation occurs, but in this case fungal burden converges to a fixed value. Our model simulation estimates that persistent or cyclic colonisation occur when the rate of fungal proliferation is lower or higher, respectively, than 0.15 h^−1^ (Fig. 7(a), black vertical line).

In all three distinct infectious scenarios (cleared, cyclic or persistent colonisation in Fig. 8), both F and N first increase until the curative threshold (Nc, dotted lines) is met, at which point fungal burden begins to decrease. Our model suggests that fungal containment requires sufficient fungal proliferation to prompt cytokine-mediated neutrophil recruitment, to a value exceeding Nc. Resolution of colonisation is achievable if F is cleared before N starts decreasing, the latter dictated by reduction of F-dependent cytokine production. If the fungal burden remains insufficiently curtailed it will proliferate once more when recruited neutrophils fall below Nc. This leads to cyclic or persistent colonisation. Note that Nc in our model varies as a function of the fungal proliferation rate. This is a counter-intuitive cause of cyclic and persistent colonisation following a single fungal exposure. Our model also predicts that cyclic and persistent colonisation also occurs in cases of repeated fungal exposure (Fig. S4).

### Mechanistic basis of cyclic and persistent colonisation

Mechanistic insights on how some combinations of initial fungal burden and the fungal proliferation rate (*β*) might lead to chronic colonisation in our model are obtainable by plotting the clearance time against fungal proliferation rate for different fungal burdens (Fig. 7(b,c)). Examination of *β* -dependent change in the clearance time during low dose inoculation (Fig. 7(b)) reveals four distinct behaviors (A, B, C and D), where (A) fungal clearance (mainly macrophage-dependent) is achieved, (B) colonisation becomes persistent or cyclic, (C) fungal clearance (mainly by neutrophils) is achieved, and (D) saturation of fungal burden is reached. For behavior (A), fungal clearance is always achieved, since macrophages are sufficient to contain the fungal challenge, irrespective of the level of recruited neutrophils. Note that Nc < 0 in this case. For (B), macrophages are no longer sufficient to clear the infection and low-level fungal colonisation occurs (persistent colonisation, Fig. 8 violet). In this case fungal persistence occurs since although the escalation of fungal burden is sufficient to elicit an immune response, it is too weak to provoke a fully curative immune response. At the B/C border *β* has become large enough to heighten immune responses resulting in cyclic colonisation (Fig. 8, cyan). For (C) the pathogen proliferates quickly enough to prompt a curative immune response to clear the fungal challenge, and for (D) the rate of fungal proliferation overwhelms the host response leading to saturation of the fungal burden, as in neutropenic hosts (Fig. 2(b)). Our nominal value for *β* (Fig. 7(b), filled circles) is almost in the middle of the region C, and not close to its neighboring regions, implying that random parameter variations in *β* are least likely to move the system into the region B or D, where clearance is not achieved. For high-dose inoculation, the region B does not exist (Fig. 7(c)), in accordance with the observations that high dose inoculation does not exhibit subclinical chronic (oscillatory) colonisation (Fig. 7(a)). However, the increase in the clearance time by decrease in the proliferation rate can still be observed for certain combinations of the initial burden and the proliferation rate (Fig. 7(c)).

## Discussion

Fungal infections are the outcome of complex host-pathogen interactions, our mechanistic understanding of which is largely based upon integration of data derived from multiple types of *in vivo* and *in vitro* experimentation. This approach is subject to fatal constraints, including limited throughput and the ethical availability of resources, thereby preventing a thorough evaluation of certain conditions in experimental systems and the likely impact of their variance upon outcome of fungal exposure. Mathematical modelling of disease can overcome these barriers by providing the opportunity to quantitatively assess the impact of variability in model systems, and to identify those parameters most potently influencing the outcome of infection. In the setting of opportunistic fungal infections, a quantitative framework to assess the relative contributions of both host and pathogen activities to the outcome of disease, is a fundamental prerequisite for understanding the mechanistic basis of chronic disease, and could be particularly useful for the implementation of curative chemotherapeutic and immunomodulatory interventions.

This study addresses diseases caused by the mould pathogen *A. fumigatus* where the outcome of spore inhalation can vary enormously between individuals, being ultimately governed by host immune status, and where experimental approaches to modelling infection fail to capture physiologically relevant host-pathogen interactions as they often require the use of high inoculation doses. Although mathematical modelling has been recently applied to simulation of responses to antifungal therapy^31^, analysis of fungal sphingolipid metabolism during pH shifts in phagolysosomes^32^, immune response against *C. albicans* in human blood^33^, and early immune response by alveolar macrophages to *A. fumigatus*^34^, a quantitative understanding of the host-pathogen interaction within the context of fungal disease in whole animals, has not yet been achieved for any fungal pathogen.

In this study we constructed a mathematical model to examine the impact of concerted actions of macrophage and neutrophils in response to *A. fumigatus* spore inhalation, using data derived from murine *in vivo*, *ex vivo* and *in vitro* experimentation (Table 1) to parameterise processes involved in healthy containment of inhaled fungal spores (Figs 1 and 2(a)). We then used the model to calculate the impact of neutrophil depletion. Concordant with risk factors for invasive aspergillosis in human subjects, *in silico* depletion of available neutrophils lead to rapid fungal pneumonia (Fig. 2(b)). The model further allowed us to quantify (i) the relative contributions of two mechanisms required for direct fungal clearance, i.e. killing by macrophages and neutrophils (Fig. 3(a)), and (ii) the impacts of variance in macrophage and neutrophil titres (Fig. 3(b)), timing and rate of neutrophil recruitment (Fig. 4) and size of infectious inoculum (Fig. 5) upon the outcome of fungal exposure.

Our results concur with the classically proposed two-step mechanism for effective fungal containment^8^, first by macrophages followed by neutrophil killing (Fig. 3(a)). Specifically, we identified a requirement for the host to satisfy a threshold value, Nc, for recruited neutrophils in order to implement fungal containment. Sufficient recruitment of neutrophils requires the production of cytokines by macrophages, explaining the indirect contribution of macrophages to fungal containment (Fig. 3(b)). The reported risk of fungal infection in patients undergoing TNF-α depletion^29^ might also be due to less efficient neutrophil recruitment by TNF-α, leading to less efficient fungal containment (Fig. 4(b)). Our *in silico* experiments also suggested the importance of N recruitment at early phases of fungal challenge (Fig. 4(d)), as previously reported from murine challenge studies^18^,^25^,^27^. Our mathematical model predicted that low dose fungal challenges persist in immunocompetent hosts, as it takes longer to achieve the threshold value Nc. The later occurrence of peak F values for lower fungal exposure was supported by the outcome of experimental fungal exposure in mice (Figs 5 and 6). The mechanistic insight derivable from our *in silico* studies suggest a wealth of immunotherapeutic options to explore in future experimentation.

Our model analysis further suggests the appearance of persistent or cyclic colonisation when the fungal proliferation rate is reduced, due to insufficient recruitment of neutrophils in low dose inoculation (Figs 7 and 8). The mechanistic basis for cyclic and persistent colonisation caused by reduction in the fungal proliferation rate can be explained by the existence of delayed negative feedback on the fungal burden through cytokine-induced neutrophil recruitment, combined with positive feedback on the fungal burden by fungal proliferation. Accordingly, the occurrence of cyclic or persistent colonisation can also be prompted *in silico* by perturbations other than the rate of fungal proliferation. For example, in response to alterations in host status (increased N recruitment rate or increased cytokine production rate) that make the negative feedback gain higher, cyclic or persistent colonisation occurs because Nc is reached too quickly, thereby resulting in early quelling of fungal burden and a consequently premature dampening of neutrophil recruitment. Oscillatory behaviors are clinically observed as chronic lung infection, where cyclical exacerbations of infection occur in non-neutropenic individuals, for example in the settings of cystic fibrosis or chronic obstructive pulmonary disease^35^.

The current model assumes that macrophages are the major source of cytokine production in both immunocompetent and neutropenic cases. This is likely to be an over-simplification. We note that the limited available evidence does not support an experimentally measurable impact of macrophage depletion upon outcome of murine aspergillosis^25^, yet insights derivable from our study reveal the exquisite sensitivity of the host to relatively modest alterations of fungal proliferation (Fig. 7). It therefore remains plausible that even apparently minor, directly or indirectly implemented, microbicidal activities have the capacity to influence outcomes of fungal ioculation. Whilst the *in silico* data presented in this study depict relative contributions of macrophages and neutrophils to high dose inocula, the dynamic response to low dose exposure is highly similar, thereby highlighting the value of mathematical simulations in extracting mechanistic information from complex biological problems. Inclusion, in our model, of further immunomodulatory molecules and cells (including natural killer cells, epithelial cells and endothelial cells) will provide better, and more quantitative, insight into the contribution of other host-derived entities to resolution of pulmonary fungal infections.

## Methods

### Mathematical models

Our model (Fig. 1) is described by a set of four ordinary differential equations as


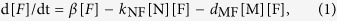



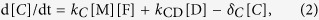



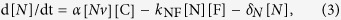



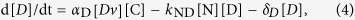


where [F], [C], [N] and [D] indicate the fungal burden [10^6^ CEs], concentrations of cytokines [ng/ml], recruited neutrophils [10^6^ cells] and iDCs [10^6^ cells], respectively. [M], [Nv] and [Dv] are the fixed concentrations of the available macrophages, available neutrophils and accessible iDCs, respectively. Note that α_D_[Dv] is estimated as a single parameter.

The 13 model parameters are summarised in Table 1 with their nominal values used for the simulation. The nominal values for the first 8 parameters in Table 1 were directly derived from previously documented murine *in vivo* or *in vitro* experimentation. The remaining 5 parameters, whose values were not directly available from experimental data, were estimated by parameter optimization to reproduce the time course data documented in the literature^20^,^24^ and our in-house experiments (see Murine challenge experiments). Details of the parameter derivation and the optimization procedures are found in Supplementary Information.

### Model analysis

All the model analysis was conducted using MATLAB version R2012a (The MathWorks, Inc., Natick, MA, USA) and R app GUI 1.62, 6558 Snow Leopard build (R Foundation for Statistical Computing, 2012). We assumed that the system is at a steady state [F] = [C] = [N] = [D] =0 before *t *= 0, the time at which inhaled spores reach the lung. The simulation was stopped when F becomes <1. Clearance time is the time required for F to become <1.

Global parameter sensitivity analysis of the proposed model was performed to identify sensitive parameters (with their respective nominal parameter values) for the time required for fungal clearance (both high dose and low dose exposures). Details of the analysis conducted are shown in Supplementary Information. The most sensitive parameter was identified to be β. M, Nv, α, kc and d_MF_ were also sensitive, confirming the importance of the balance between the fungal proliferation and capabilities of direct killing of fungal burden by macrophages and neutrophils, recruited through cytokine production. We further conducted numerical analysis to confirm that the model predictions shown in this study are robust to parameter perturbation (detailed in Supplementary Information).

### Murine challenge experiments

Murine exposures to fungal spores were performed under UK Home office project Licence PPL/70/7324 in dedicated facilities at Imperial College London using methodologies approved by the UK Home Office and the Ethical Review Panel of Imperial College London. All experiments were carried out in accordance with these approvals, and in accordance with the Good Practice guidelines laid down by the Laboratory Animal Science Association (http://www.lasa.co.uk/). Outbred male mice (strain CD1, 18–22 g, Harlan Ortech) were housed in individually vented cages. Mice were immunosuppressed using cyclophosphamide injection, via the intraperitoneal route, at a dose of 150 mg/kg, on days −3, and −1 relative to inoculation, and at subsequent 3 day intervals. A single subcutaneous dose of hydrocortisone acetate was administered on day −1. *A. fumigatus* spores for inoculation were grown on *Aspergillus* complete medium, containing 1% (v/v) glucose and 5 mM ammonium (+)-tartrate for 5 days prior to inoculation. Conidia were freshly harvested using sterile saline (Baxter Healthcare) and filtered through Miracloth (Calbiochem). Conidial suspensions were spun for 10 minutes at 3000 *g*, washed twice with sterile saline, counted using a hemocytometer and re-suspended at the relevant concentrations. Viable counts from administered inocula were determined following serial dilution by plating on *Aspergillus* complete medium containing 1% (v/v) glucose and 5 mM ammonium (+)-tartrate and growth at 37 °C. Mice were anaesthetized by isofluorane inhalation and infected by intranasal instillation.

### Quantification of fungal burden

For enumeration of colony forming units (CFUs) mice were sacrificed at the relevant time points post-inoculation, lungs removed, homogenised in sterile saline and serially diluted prior to culture on ACM plates supplemented with 1% (w/v) glucose and 5 mM ammonium tartrate. ACM plates were incubated for 3 days at 37 °C prior to enumeration of CFUs. For enumeration of conidial equivalents (CEs), quantitative PCR (qPCR) was performed on total genomic DNA extracted, using the DNeasy Blood and Tissue Kit (Qiagen), from homogenized whole lungs. Fungal burden was assessed by quantitative PCR amplification using the oligonucleotides BT-F (GAGCCCTTTTCCGACCTGAT) and BT-R (GGAACTCCTCCCGGATCTTG) to amplify the beta tubulin (AFUA_7g00250) locus from the *A. fumigatus* genome. qPCR was performed using Sybr Green Jumpstart™Taq ReadyMix™ (Sigma-Aldrich, UK). Standard curves for fungal burden were generated by ‘spiking’ gDNA extracted from known numbers of *A. fumigatus* conidia into gDNA extracted from uninfected lung homogenates (see Supplementary Information for raw data). The experiment was performed as four technical replicates. For all samples returning a minimum of n = 2 replicate data points, data were included in the analysis. Raw replicate data are presented in Table S2.

### Mathematical model for dead fungal cells

While our model (1)–(4) considers only live fungal cells*, in vivo* experiments using qPCR measures nuclear equivalents (CEs), which can derive from both live and dead fungal cells. In order to mitigate over-quantification of live fungi, we define the total fungal burden, measured by qPCR, as *F*_all _= *F *+ *F*_*d*_ , where *F*_*d*_ corresponds to dead fungal cells and might correspond to fungi which have been killed by neutrophils or macrophages, but have yet to be cleared. qPCR data is compared to the predicted dynamics of *F*_all_, where the dynamics of *F*_*d*_ is described by d[*F*_*d*_]/dt = *k*_NF_[N][F]+ *d*_MF_[M][F] − *δ*_*F*_[Fd]. The parameter value for *δ*_*F *_= 0.090 was derived by fitting the CE measurement data, *F*_all_(48) = 3.1380 and *F*_all_(65) = 0.6807, for exposure to 4.25 × 10^6^ CEs *A. fumigatus* in immunocompetent mice (Fig. 6(b)), to the equation *F*_all_(*t*) = *F*_all_(48)exp(*−δ*_*F*_(*t-*48)). This equation is derived based on the assumption that the live fungal cells *F* is very low at 48 hours after this high-dose exposure (Fig. 2(a)), and that the decay of *F*_all_ at t >48 hours is due to the clearance of F_d_.

## Additional Information

**How to cite this article**: Tanaka, R. J. *et al.* *In silico* modeling of spore inhalation reveals fungal persistence following low dose exposure. *Sci. Rep.* **5**, 13958; doi: 10.1038/srep13958 (2015).

## Supplementary Material

Supplementary Information

## Figures and Tables

**Table 1:** Definitions of model parameters and their nominal values.

**Parameters**		**Nominal values [unit]**	**Ref.**
*M*	Macrophages	0.3 [10^6^ cells]	36
*N*v	Total Neutrophil pool	150 [10^6^ cells]	37
*δ*_*N*_	N degradation rate	6.1 × 10^−2^ [h^−1^]	38
*δ*_*C*_	C degradation rate	6.6 × 10^−2^ [h^−1^]	28
*k*_ND_	N-induced D efflux rate	6.9 × 10^−3^ [10^−6^ cells^−1^ h^−1^]	22
*k*_NF_	N-mediated F killing rate	1.2 [10^−6^ cells^−1^ h^−1^]	10
*d*_MF_	M-mediated F killing rate	3.2 × 10^−1^ [10^−6^ cells^−1^ h^−1^]	9,18
*β*	F proliferation rate	2.8 × 10^−1^ [h^−1^]	39
*α*	N recruitment rate	1.7 × 10^−3^ [ml ng^−1^ h^−1^]	
*α*_D_[*D*v]	D recruitment rate	1.7 × 10^−2^ [ml 10^6^ cells ng^−1^ h^−1^]	
*k*_CD_	D-induced C production rate	3.1 ×10^−1^ [ml^−1^ 10^−6^ cells^−1^ ng h^−1^]	
*k*_C_	M-induced C production rate	3.8 × 10^−1^ [ml^−1^ 10^−6^ cells^−1^ ng h^−1^]	
*δ*_*D*_	D degradation rate	1.0 × 10^−1^ [h^−1^]	
