# Supplementary material for: In silico modeling of spore inhalation reveals fungal persistence following low dose exposure
Source: Sci Rep. 2015 Sep 14;5:13958. doi: 10.1038/srep13958 (PMC4568477; doi:10.1038/srep13958)
Supplement: Supplementary Information [file srep13958-s1.pdf]

In silico modeling of spore inhalation reveals fungal persistence following low dose exposure
Supplementary Information

Reiko J. Tanaka,* Neville J. Boon, Katarina Vrcelj, Anita Nguyen, Carmelina Vinci,

Darius Armstrong-James, and Elaine Bignell†

1 Parameter estimation

Our model described by Equations (1)-(4) in the main text is comprised of 13 parameters (Table 1), whose nominal values were estimated either by direct derivation from previously documented *in vitro* and *in vivo* experimentation, or by parameter optimization to reproduce the dynamic behaviors for variables F, C, N and D observed in experimental murine exposure to *A. fumigatus* spores in [1, 2] and in our own experimentation.

1.1 Direct derivation of the parameters

The first 8 parameters ($M, N_v, \delta_N, \delta_C, \delta_F, k_{ND}, k_{NF}, d_{MF}$ and β) listed in Table 1 were directly derived from the experimental data as described below. We use 2 significant digits by rounding the third digit.

Number of macrophages M. Hohl et al. [3] measured the absolute number of macrophages in murine bronchoalveolar lavage fluid (BAL), at 24 hours post-inoculation with an intranasal inoculum of 10^7 live *A. fumigatus* conidia. In our model we assume that the number of macrophages remains constant and we use the observed value of $M = 0.3 \times 10^6$ cells (Fig. 1 in [3]).

Number of available neutrophils N_v. Assuming total murine blood volume to be 58.5ml/kg, the circulating volume of blood in a mouse with a typical weight of 25 g can be estimated as $58.5 \times 25 \times 10^{-3} = 1.4625$ ml. If the total number of white blood cells average, in mice 7.45×10^3 cells/μl (Fig. 3 in [4]), of which 14.63% are neutrophils [4, 5], the circulating neutrophil titre can be calculated as $7.45 \times 10^3 \times 0.1463 \times 1.4625 \times 10^3 = 1.59 \times 10^6$ cells. Since circulating neutrophil count is estimated as accounting for $1-2\%$ of the total body neutrophil count [5], we obtain $N_v = 150 \times 10^6$ cells as a number of the total neutrophils available.

N-mediated F killing rate k_{NF}. *In vitro* incubation of *A. fumigatus* spores (10^5 ml^{-1}) with an equal number of neutrophils lead to killing of 30.6% of the spores after 2-3 hours (Table1 in [6]). Assuming the rate of change of the number of spores F in this setting is described by

$$\frac{dF}{dt} = -k_{NF}NF$$

*r.tanaka@imperial.ac.uk

†elaine.bignell@manchester.ac.uk

without fungal proliferation, we obtain $k_{NF} = 1.2176 \approx 1.2 \times 10^{-6}$ cells^{-1}h^{-1}.

M-mediated F killing rate d_{MF}**.** *In vitro* challenge of cultured human alveolar macrophages (0.5×10^6 cells/coverslip) with *A. fumigatus* conidia (two conidia per macrophage) resulted in engulfment of 90 % of bound *A. fumigatus* conidia after 18 hours (Fig. 7 in [7]). Assuming the rate of change of fungal burden F in this setting is described by

$$\frac{dF}{dt} = -d_{MF}MF,$$

we obtain $d_{\mathrm{MF}} = 0.4264 \times 10^{-6}$ cells^{-1}h^{-1}.

Ex vivo killing assay found 47 % of *A. fumigatus* swollen conidia were killed after 6 hours of incubation with 0.5×10^6 murine alveolar macrophages (Fig. 4 in [8]). In this study alveolar macrophages were harvested from mouse lungs and seeded at a conidium/AM ratio of 1:10. We therefore derived $d_{\mathrm{MF}} = 0.2116 \times 10^{-6}$ cells^{-1}h^{-1}.

We therefore chose the nominal value of $d_{\mathrm{MF}} = 0.32 \times 10^{-6}$ cells^{-1}h^{-1} by taking the mean of these two values.

F proliferation rate β**.** Vallor *et al.* [9] measured the temporal change of *A. fumigatus* fungal burden in a neutropenic guinea pig model of invasive pulmonary aspergillosis. We assume the fungal proliferation rate is approximate to that in a mouse. From their data (Table 1) $F(1\mathrm{h}) = 10^{5.9}$ conidial equivalents (CEs) and $F(24\mathrm{h}) = 10^{7.7}$ CEs.

The model equation

$$\frac{dF}{dt} = (\beta - d_{MF}M)F,$$

$\beta = 2.8 \times 10^{-1} h^{-1}$ is derived since $\beta - d_{\mathrm{MF}}M = \dfrac{1}{(24-1)} \ln\left(\dfrac{10^{7.7}}{10^{5.9}}\right) = 0.1802$ h^{-1}, $d_{\mathrm{MF}} = 0.3189 \times 10^{-6}$ cells^{-1}h^{-1} and $M = 0.3 \times 10^6$ cells from the derivation above.

Note also that the *in vitro* growth rates of $0.2349 - 0.3375$ h^{-1} measured by a microbroth method for nine *A. fumigatus* isolates (Table 1 in [10]).

N-induced D efflux rate k_{ND}**.** The numbers of DCs in the lung (D) and the lymph node (D_{lymph}) in immunocompetent mice challenged with *A. fumigatus* were measured over 24 hours (Fig. 1B in [11]): $D_{\mathrm{lymph}}(0\mathrm{h}) = 2.5 \times 10^4$ cells and $D_{\mathrm{lymph}}(24\mathrm{h}) = 5 \times 10^4$ cells. Assuming that the increase in the lymph node DCs is due to migration of DCs from the lungs to the lymph nodes and that $N(t) \approx 1.5 \times 10^6$ cells and $D(t) \approx 0.1 \times 10^6$ cells for the first 24 hours post-inoculation (Fig. 1 in [2]) for simplification, we derived $k_{\mathrm{ND}} = 6.9 \times 10^{-3} \times 10^{-6}$ cells^{-1}h^{-1} from

$$\frac{dD_{\mathrm{lymph}}}{dt} = k_{\mathrm{ND}}ND.$$

N degradation rate δ_{N}**.** Basu *et al.* [12] measured BrdU-labeled neutrophils in murine blood at 96 and 120 hours after BrdU injection, (Fig. 5C) and calculated $\delta_{\mathrm{N}} = 0.0609 \approx 6.1 \times 10^{-2}$ h^{-1} from the rate of loss of labeled neutrophils, using the formula

$$N = N_0 e^{-\delta_{\mathrm{N}} t}.$$

time t	24
$F(t)$ (in this study)	0.2314

time t	4	8	12	24	48	72	96
$C(t)$[1]	0.2	1.4	2.85	3	1.35	0.2	0.1

time t	24	48	72
$N(t)$[2]	3	8	5

time t	24	72
$D(t)$ [2]	0.3	0.1

Table S1: The experimental data used for the parameter optimization.

This is the value we use in our model. The same method with prior published data of human and mouse neutrophil decay deduces similar values of 0.033 h^{-1} (Fig. 1 in [13]) and 0.027 h^{-1} (Fig. 2 in [14]). Note also that neutrophils have a similar lifetime in the circulation as in the tissue (≈ 0.015 h^{-1} by Fig. 2b in [15]).

C degradation rate δ_C. Mehrad *et al.* [16] measured lung TNF-α levels in cyclophosphamide-treated (immunocompromised) mice after *A. fumigatus* challenge. We assume that the decrease of the lung TNF-α level after its peak at 48 hours post-inoculation is mainly due to natural degradation and derived $\delta_C = 0.0655 \approx 6.6 \times 10^{-2}$ h^{-1} using their data $C(48\text{h}) = 5.8$ ng/ml and $C(96\text{h}) = 0.25$ ng/ml (Fig. 2B) with

$$\frac{dC}{dt} = -\delta_C C.$$

The assumption of $\delta_C C(t) >> k_{CD} D(t), k_C MF(t)$ in the model equation (2) was confirmed to be valid in our model simulation.

1.2 Parameter optimization

The remaining 5 model parameters in our model (α, $\alpha_D[D_v]$, k_{CD}, k_C and δ_D) were estimated by parameter optimization using Monte Carlo Simulated Annealing [17] to fit to the *in vivo* experimental data, while the directly evaluated 8 parameters in the previous section were fixed. The *in vivo* experimental data used for the parameter optimization (Table S1) includes the dynamics of F (CFU counting) as measured in our own murine fungal challenge experiments (see Methods in the main text and Fig. 6 (a)), C (in BAL) measured at different times after a challenge by $6 \times 10^{6.25}$ CFU of *A. fumigatus* infection (Fig. 1 in [1]), and N and D measured post challenge by killed Aspergillus hyphae ($5-9 \times 10^5$ CFU) in mice (Fig. 1A and 4B in [2]). Note that high inocula (order of 10^6 CEs) were used in these published experiments. We optimized the parameter values from 1000 randomly chosen initial values within the range of $[0,1]$ for each parameter and selected the set with the minimal error, $S = \sum_{i=1}^{13}(R_i^{\text{sim}} - R_i^{\text{exp}})^2$ between the experimentally measured value R_i^{exp} and its simulated value R_i^{sim}, as our nominal parameters.

2 Parameter sensitivity analysis

We performed sensitivity analysis to check whether our main results are robust against the perturbation in the nominal parameter values.

2.1 Global sensitivity analysis

We evaluated the global parameter sensitivity of our model, in order to identify the sensitivity of the time for N_C (and the maximum fungal burden) to be reached ($T_{F_0}^c$), and the time required for clearance (to below

one CE) of the initial fungal burden F_0 (τ_{F_0}), to simultaneous variations in the parameters. For this purpose, we performed simulations of our model for $100,000$ sets of the parameters, where the value of each of the 13 parameters was chosen from a uniform distribution of up to 10% variation around its nominal value, and derived Sobol's global sensitivity metric [18] for both a high dose ($F_0 = 10^6$ CE) and a low dose ($F_0 = 10^2$ CE) inoculation. The parameter sets for which the model does not achieve fungal clearance within 150 hours were eliminated from the analysis ($\approx 4\%$ of the $100,000$ parameter sets investigated).

Sobol's global sensitivity metrics for both $F_0 = 10^6$ and $F_0 = 10^2$ indicate that the results are most sensitive to β (the fungal growth rate), followed by M, α, Nv, k_C, d_{MF} and δ_C (Fig. S1). We confirmed the convergence of the sensitivity indices with simulations of another 100000 sets of parameters. For the low dose inoculation, β has a higher relative sensitivity than for the high dose inoculation, since the early-time dynamics is dominated by the exponential growth of the fungus (the $\beta[F]$ term in our equations) that becomes exaggerated as the initial inoculum is decreased. Indeed our results demonstrate that a slight change in β for a low dose inoculation leads to qualitatively different disease outcomes (Fig. 8). For the high dose inoculation, M has a higher relative sensitivity than for the low dose infection, since high dose spores have to be killed quickly to avoid its saturation.

2.2 Persistence of low dose fungal exposure

Our model predicted (Fig 5(a)) and the experiments verified (Fig 6(b), (c)) that longer time is required to achieve N_c for lower inoculum. To confirm whether this model prediction robustly holds despite parameter variations, we evaluated $T^c_{10^2}$ and $T^c_{10^6}$, where $T^c_{F_0}$ is the time to achieve N_c with the initial fungal burden F_0, for increasing levels of variations up to 0.1, 0.2, 0.5 and 1 orders of magnitude from the nominal values for all the parameters. The results confirmed that $T^c_{10^2} > T^c_{10^6}$ robustly holds (Fig. S2(a)). The same analysis confirmed that $\tau_{10^2} > \tau_{10^6}$ robustly holds despite parameter variations, when the curative neutrophil threshold $N_C > 0$ (Fig. S2 (b)). Note that $\tau_{10^2} < \tau_{10^6}$ are observed if $N_C < 0$, since the fungal burden does not grow initially (when $N = 0$) under this condition and thus is rapidly cleared mainly by macrophages (M), corresponding to the behaviour A in Fig. 8(b)-(c).

2.3 Existence of oscillatory behaviors

The clearance time against varied values of β for different initial fungal burdens (Fig. 8) indicates a qualitative change at $\hat{F}_0 = 10^4$, above which the region B disappears, suggesting that the oscillatory behaviors are observed only for initial fungal burden F_0 below this threshold burden $\hat{F}_0$. Our extensive set of simulations and mathematical analysis shows that the existence of the oscillatory behaviors in low dose infection is always observed for perturbation of all the parameters for at least up to an order of magnitude from the nominal values, except for the case with $N_C < 0$ corresponding to the behaviour A in Fig. 8(b)-(c).

3 Experimental data

The experimental data that we discuss in the main text is presented in Tables S1 and S2.

References

[1] Brieland JK, et al. (2001) Cytokine networking in lungs of immunocompetent mice in response to inhaled Aspergillus fumigatus. *Infection and immunity* 69:1554–1560.

[2] Park SJ, et al. (2010) Neutropenia enhances lung dendritic cell recruitment in response to Aspergillus via a cytokine-to-chemokine amplification loop. *Journal of immunology (Baltimore, Md. : 1950)* 185:6190–6197.

[3] Hohl TM, et al. (2005) Aspergillus fumigatus Triggers Inflammatory Responses by Stage-Specific β-Glucan Display. *PLoS pathogens* 1:9.

[4] Peters DG, Zhang XC, Benos PV, Heidrich-O'Hare E, Ferrell RE (2002) Genomic analysis of immediate/early response to shear stress in human coronary artery endothelial cells. *Physiol. Genomics* 12:25–33.

[5] von Vietinghoff S, Ley K (2008) Homeostatic regulation of blood neutrophil counts. *Journal of immunology (Baltimore, Md. : 1950)* 181:5183–5188.

[6] Levitz SM, Diamond RD (1985) A rapid colorimetric assay of fungal viability with the tetrazolium salt MTT. *The Journal of infectious diseases* 152:938–945.

[7] Ibrahim-Granet O, et al. (2003) Phagocytosis and intracellular fate of Aspergillus fumigatus conidia in alveolar macrophages. *Infection and immunity* 71:891–903.

[8] Philippe B, et al. (2003) Killing of Aspergillus fumigatus by alveolar macrophages is mediated by reactive oxidant intermediates. *Infection and immunity* 71:3034–3042.

[9] Vallor AC, et al. (2008) Assessment of Aspergillus fumigatus burden in pulmonary tissue of guinea pigs by quantitative PCR, galactomannan enzyme immunoassay, and quantitative culture. *Antimicrobial agents and chemotherapy* 52:2593–2598.

[10] Paisley D, Robson GD, Denning DW (2005) Correlation between in vitro growth rate and in vivo virulence in Aspergillus fumigatus. *Med.Mycol.* 43:397–401.

[11] Park SJ, Burdick MD, Mehrad B (2012) Neutrophils Mediate Maturation and Efflux of Lung Dendritic Cells in Response to Aspergillus fumigatus Germ Tubes. 80:1759–1765.

[12] Basu S, Hodgson G, Katz M, Dunn AR (2002) Evaluation of role of G-CSF in the production, survival, and release of neutrophils from bone marrow into circulation. *Blood* 100:854–861.

[13] Lord BI, et al. (1991) Myeloid cell kinetics in mice treated with recombinant interleukin-3, granulocyte colony-stimulating factor (CSF), or granulocyte-macrophage CSF in vivo. *Blood* 77:2154–2159.

[14] Price TH, Chatta GS, Dale DC (1996) Effect of recombinant granulocyte colony-stimulating factor on neutrophil kinetics in normal young and elderly humans. *Blood* 88:335–340.

[15] Kim MHH, et al. (2008) Dynamics of neutrophil infiltration during cutaneous wound healing and infection using fluorescence imaging. *The Journal of investigative dermatology* 128:1812–20.

[16] Mehrad B, Strieter RM, Standiford TJ (1999) Role of TNF-alpha in pulmonary host defense in murine invasive aspergillosis. *Journal of immunology (Baltimore, Md. : 1950)* 162:1633–1640.

[17] Kirkpatrick S, Gelatt CD, Vecchi MP (1983) Optimization by simulated annealing. *Science (New York, N.Y.)* 220:671–680.

[18] Sobol IM (2001) Global sensitivity indices for nonlinear mathematical models and their Monte Carlo estimates. *Mathematics and Computers in Simulation* 55:271–280.

4×10^6 CFUs	400 CFUs
113000	90
209000	50
220000	120
320000	10
295000	40

Table S1: CFUs at 24 hours post-inoculation, per whole murine lung, following intranasal inoculation of immunocompetent CD1 mice with 4×10^6 or 400 *A. fumigatus* colony forming units (CFUs). CFU counts were determined by culture of homogenised lungs on ACM agar, and enumeration of colonies.

4.25×10^6 CFUs			4.25×10^4 CFUs		
24 hours	48 hours	65 hours	24 hours	48 hours	65 hours
397348	3091148	312913	157628	60599	237843
7087955	518688	489316	39710	900629	23264
3466755	244377	371865	199202	45370	60357
367069	409021	735221	1049977	100448	234624
547264	6261505	628365	834470	229045	210952
7060366	3226818	824466	295442	387174	120926
288312	609715	313049	125002	514299	31988
397138	16421170	564623	116275	378004	162268
280937	1875676	2092821	540132	120633	320754
11317003	240354	568106	97729	58768	281695
13357176	569908	586725	342645	225346	57873
2646809	665083		428817	82693	43922
8256643	11319847		324899	29425	
24264970	1195520		145906	528250	
	420585		33778	1725395	
			120057	1478676	
			110742	89312	
			32384	86707	
			45110		

Table S2: Conidial equivalents (CEs) at 24, 48 and 65 hours post-inoculation, per whole murine lung, following intranasal inoculation of immunocompetent CD1 mice with 4.25×10^6 or 4.25×10^4 *A. fumigatus* colony forming units (CFUs). CE counts were derived by qPCR-mediated quantitiation of the *A. fumigatus* β-tubulin gene. Standard curves (CT) of the form $CT = A \log_{10}(CE) + B$ were derived from spiked genomic DNA (gDNA) samples by combining gDNA from known quantities of *A. fumigatus* spores ($10^2 - 10^6$) with gDNA extracted from uninfected murine lungs. CEs were estimated by interpolation from the standard curves using $(A, B) = (-2.2725, 48.6679)$ for 4.25×10^6 and $(A, B) = (-2.1206, 45.6605)$ for 4.25×10^4 CEs.

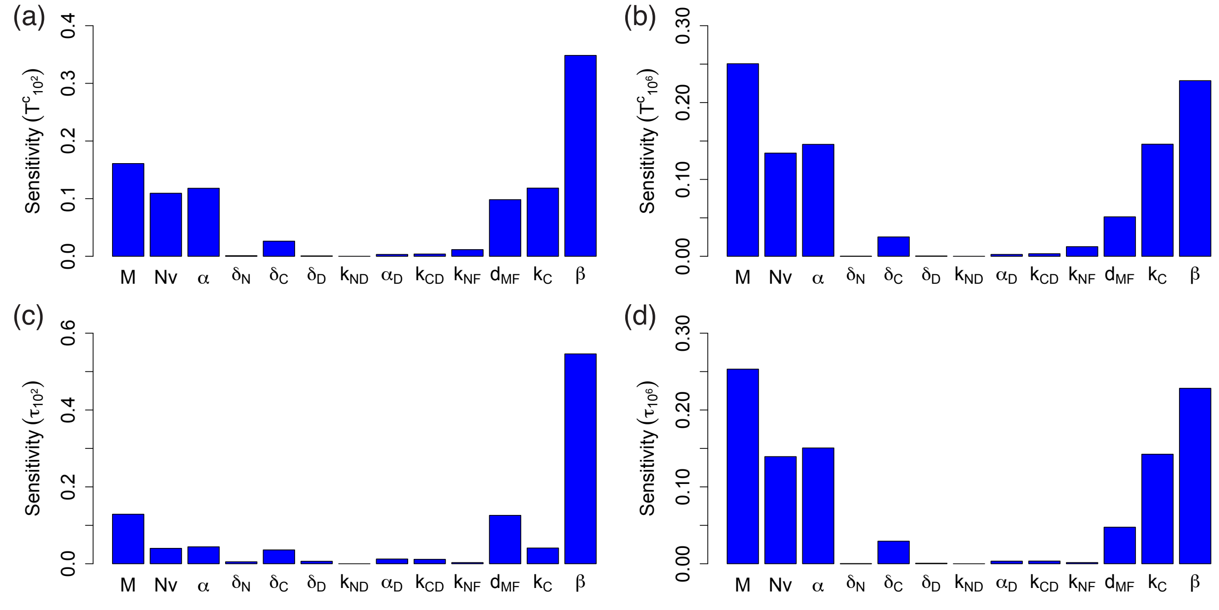

Figure S1: SOBOL global sensitivity analysis for (a) $T^c_{10^2}$, (b) $T^c_{10^6}$, (c) τ_{10^2} and (d) τ_{10^6}.

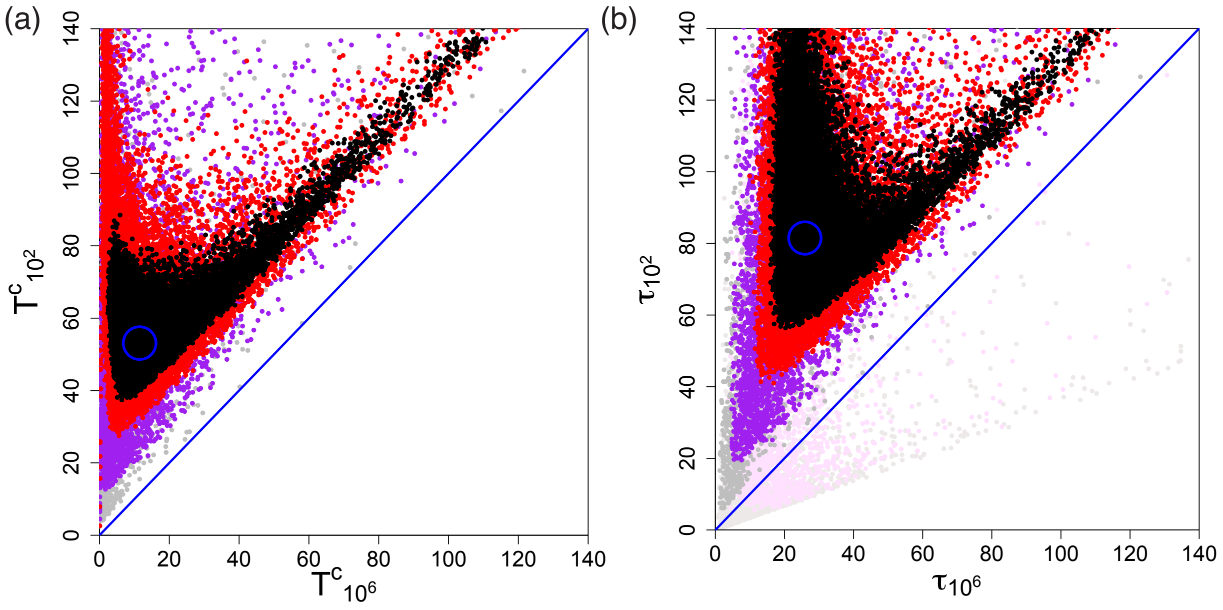

Figure S2: Simulation results for uniformly sampled parameter sets, each of which deviates up to 0.1 (black), 0.2 (red), 0.5 (purple/light pink) or 1 (grey/light grey) order of magnitude from its nominal value (blue circle). (a) $T^c_{10^2}$ against $T^c_{10^6}$ for the cases N_C is reached. All results lie above the blue line suggesting that $T^c_{10^2} > T^c_{10^6}$. (b) τ_{10^2} against τ_{10^6}. Light pink and light grey points represent parameter sets where $N_C < 0$. For $N_C > 0$, when the neutrophils are required for clearance, all results lie above the blue line meaning that $\tau_{10^2} > \tau_{10^6}$.

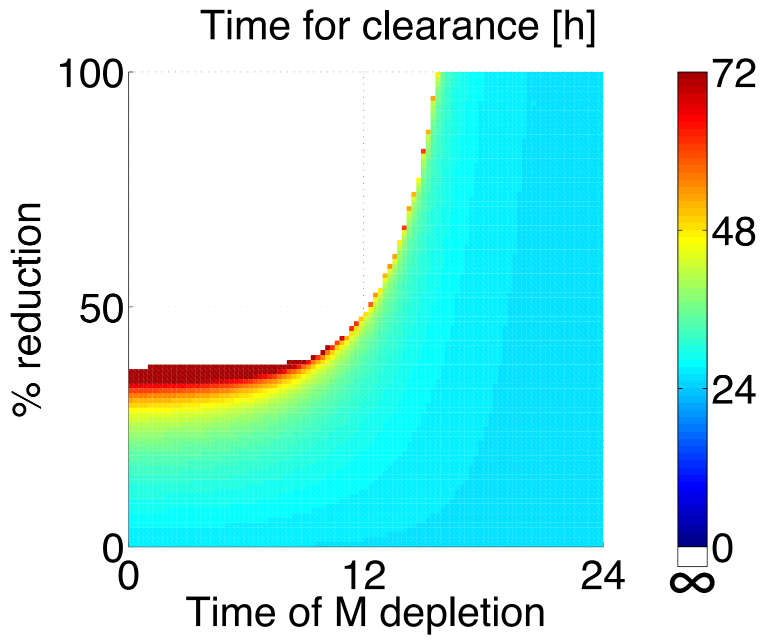

Figure S3: Time for fungal clearance [h] with varied rate of reductions and timings of reduction introduced (in hours post-inoculation) for M.

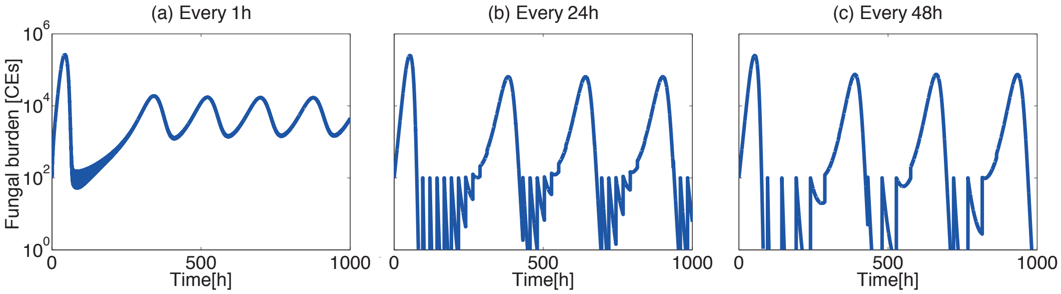

Figure S4: Model prediction of cyclic and persistent colonisation following repeated fungal exposure of 100 CEs every (a) 1h, (b) 24h and (c) 48h. Rapid changes in the fungal burden (described by a thicker line) are observed between 50 and 300 hours in (a).
